# Protective Effect of Cyanidin-3-*O*-Glucoside against Ultraviolet B Radiation-Induced Cell Damage in Human HaCaT Keratinocytes

**DOI:** 10.3389/fphar.2016.00301

**Published:** 2016-09-07

**Authors:** Yunfeng Hu, Yuetang Ma, Shi Wu, Tianfeng Chen, Yong He, Jianxia Sun, Rui Jiao, Xinwei Jiang, Yadong Huang, Liehua Deng, Weibin Bai

**Affiliations:** ^1^Department of Dermatology, The First Affiliated Hospital of Jinan University, Jinan UniversityGuangzhou, China; ^2^Department of Food Science and Engineering, Jinan UniversityGuangzhou, China; ^3^Faculty of Chemical Engineering and Light Industry, Guangdong University of TechnologyGuangzhou, China

**Keywords:** Cyanidin-3-*O*-glucoside, HaCaT keratinocytes, ultraviolet B radiation, reactive oxygen species, apoptosis

## Abstract

Ultraviolet radiation is the major environmental harmful factor that has emotional impact on human skin. The aim of the present study was to determine the mechanism of protection of cyanidin-3-*O*-glucoside against ultraviolet B (UVB)-induced damage to human HaCaT keratinocytes. Our results show that cyanidin-3-*O*-glucoside decreased the levels of intracellular reactive oxygen species generated by UVB treatment. Cyanidin-3-*O*-glucoside also decreased the UVB-augmented levels of the DNA damage indicators phospho-p53 and phospho-ATM/ATR. In addition, cyanidin-3-*O*-glucoside protected keratinocytes from UVB-induced injury by overturning the disruption of mitochondrial membrane potential and reversing apoptosis. The expression of anti-apoptotic protein B-cell lymphoma 2 (Bcl-2) was attenuated in UVB-exposed cells but restored in UVB/cyanidin-3-*O*-glucoside-treated cells. Furthermore, expression of the proapoptotic proteins Bcl-2-associated X (Bax) and the key apoptosis executer cleaved caspase-3 were increased in UVB-irradiated cells and decreased in UVB/cyanidin-3-*O*-glucoside-treated cells. For these reasons, the results demonstrate that cyanidin-3-*O*-glucoside protects human keratinocytes against UVB-induced oxidative stress and apoptosis. Our study provides a theoretical basis for the use of cyanidin-3-*O*-glucoside in the fight against light damage.

## Introduction

Ultraviolet (UV) radiation from the sun induces several harmful responses, including erythema, immunosuppression, edema, sunburn, hyperplasia, hyperpigmentation, premature aging, and skin cancer (de Gruijl et al., 1993; Fisher et al., 1997; Viertler et al., 2012). Only UVA (320–400 nm) and UVB (280–320 nm) are harmful to human skin. UVA accounts for more than 90% of the total UV radiation reaching the earth’s surface and is constant throughout the year. UVB photonsare one thousand times more capable of causing sunburn than UVA and increase considerably in the summer (Svobodova et al., 2003). UVA is considered to play a crucial role in photoaging and causes epidermal hyperplasia, stratum corneum thickening, and synthesis of inflammatory cytokines and matrix metalloproteinases (MMPs) (Wlaschek et al., 1994; Lavker et al., 1995). UVB causes sunburn, sun tanning, pigmented spots, wrinkles, and accelerates skin aging (Lavker et al., 1995).

Ultraviolet B radiation induces pivotal reactive oxygen species (ROS) in the skin and cultured skin cells, leading to gene mutations and abnormal cellular proliferation (Hattori et al., 1996; Ahmed et al., 1999). These ROS are generated by transferring electromagnetic energy from UVB radiation to molecular oxygen. At least 50% of the damage caused by UVB light is due to ROS formation (Dinkova-Kostova, 2008). UVB radiation as a potent ROS inducer can permeate through the epidermis to the dermis, contributing to skin wrinkling, freckling, and the development of skin cancers (Amaral et al., 2013; Lee et al., 2013).

Major protective systems in human skin cells are the natural pigment melanin which absorbs and scatters UV radiation, and antioxidant enzymes (catalase, superoxide dismutase, and glutathione peroxidase) (Yaar and Gilchrest, 2007). Moreover, p53 protein is also involved in UVB protection, acting as a transcription factor that controls genes in the cell cycle, apoptosis, and DNA repair (Kang et al., 2008).

Human skin without protection is inadvertently exposed to approximately two-thirds of the cumulative erythemal UV dose/year (Sies and Stahl, 2004). Therefore, next to sunscreen protection when consciously exposed (Latha et al., 2013), photoprotection by dietary compounds via endogenous delivery to the skin may significantly contribute to lifelong protection of skin health. Phytochemicals have been reported to be effective in preventing UV-induced DNA oxidative damage through a ROS scavenging mechanism *in vitro* and in animal models (Vostalova et al., 2010). Rosmarinic acid against UVA-induced changes in a human keratinocyte cell line (HaCaT) (Psotova et al., 2006) and inhibit cutaneous alterations (skin photocarcinogenesis) caused by UVA exposure in B16 melanoma cells and mice (Sanchez-Campillo et al., 2009). Silibin from milk thistle (Singh and Agarwal, 2005) have exhibited skin protective effects against UVB-induced ROS in mouse and cell models through p53 activation and decreased DNA damage. Chrysin attenuates apoptosis, ROS generation, and cyclooxygenase 2 expression and diminishes down-regulation of aquaporin-3 induced by UVB and UVA (Wu et al., 2011; Murapa et al., 2012).

Dietary guidelines around the world recommend increased consumption of fruits and vegetables as good sources of antioxidant phytochemicals for the prevention of chronic diseases (Rao and Snyder, 2010). The anthocyanin cyanidin-3-*O*-glucoside and other phytochemicals are recognized as potent antioxidants and free radical scavengers (Speciale et al., 2010; Sun et al., 2010, 2011a,b). Their scavenging capacity against free radicals and other ROS are used in the management of many chronic diseases including diabetes mellitus, atherosclerosis, cancer, and cardiovascular disease (Sato et al., 1999; Bailey et al., 2000). To the best of our knowledge, little is known about the protective effects of cyanidin-3-*O*-glucoside against UVB radiation-induced damage in skin keratinocytes. Therefore, in the present study, we aimed to investigate the ability of cyanidin-3-*O*-glucoside to protect human HaCaT keratinocytes from UVB-induced oxidative stress and apoptosis.

## Materials and Methods

### Reagents

Cyanidin-3-*O*-glucoside [purity: ≥ 98% (HPLC)] was purchased from Chengdu Stegmann Biotechnology Co., Ltd., China. MTT reagent and DCFH-DA probe were purchased from Sigma. The mitochondrial membrane potential detection reagent kit (JC-1) and apoptosis detection reagent kit were Biyuntian Biotechnology Research Institute products. The UVB lamp was manufactured by Philips. HaCaT cells were a gift from the College of Life Science and Technology, Jinan University.

### Cell Culture

The human keratinocyte cell line, HaCaT, was maintained at 37°C in an incubator with a humidified atmosphere of 5% CO_2_. The cells were cultured in Dulbecco’s modified Eagle’s medium (Gibco, Rockville, MD, USA) containing 10% heat-inactivated fetal calf serum, streptomycin (100 μg/mL), and penicillin (100 U/mL).

### Treatment of Cells, UVB Irradiation, and MTT Survival Assay

Cells were cultured in 96-well plates and maintained in medium for 24 h. For treatments, cells at 50–70% confluence were washed with phosphate-buffered saline (PBS) before treatment with UVB light at 100–400 mJ/cm^2^. Thereafter, PBS was replaced with fresh medium, and the cells were incubated for 12 h for viability assays or 2 h for ROS assays. The MTT assay was used for determining cellular viability (Carmichael et al., 1987). The cell protection level was calculated as the percentage of cell viability recovered under a certain condition, where 100% was defined as the difference between non-irradiated cells and irradiated cells in the absence of the extract.

### Detection of Intracellular ROS

The DCFH-DA assay was used to detect intracellular ROS generated by UVB in UVB-treated HaCaT cells (Rosenkranz et al., 1992). The cells were seeded into 96-well plates at a density of 1.0 × 10^6^ cells/mL and treated with UVB (300 mJ/cm^2^) and the followed cyanidin-3-*O*-glucoside. After 1 h incubation at 37°C, the medium was replaced with fresh medium, and the plates were again incubated for 11 h at 37°C. A DCFH-DA solution (50 μM) was then added to the cells. The fluorescence of the 2′,7′-dichlorofluorescein product was detected and quantified using a PerkinElmer LS-5B spectrofluorometer after 10 min.

### Phosphatidylserine Exposure with Annexin V-Fluorescein Isothiocyanate (FITC) Staining

Staining was performed according to the kit manufacturer’s instructions (BD Biosciences, USA). Briefly, cells were resuspended in 200 mL binding buffer and incubated with 5 mL Annexin V-FITC for 30 min at room temperature. Thereafter, 10 mL propidium iodide was added, and stained cells were examined using a Partec PAS III flow cytometer.

### Analysis of Mitochondrial Membrane Potential

Cells were exposed to UVB radiation (300 mJ/cm^2^), treated with cyanidin-3-*O*-glucoside, and incubated at 37°C for 12 h. They were then stained with JC-1 (5 μM) to detect mitochondrial polarity and analyzed by flow cytometry and confocal microscopy with the laser scanning microscope 5 PASCAL program.

### Western Blot Analysis

Harvested cells were lysed by incubation on ice for 10 min in 150 μL lysis buffer (120 mM NaCl, 40 mM Tris (pH 8), and 0.1% NP-40). The cell lysates were then centrifuged at 13,000 × *g* for 5 min. The supernatants were collected, and protein concentrations were determined. Aliquots of the lysates (15 μg protein) were boiled for 5 min and electrophoresedon a 12% sodium dodecyl sulfate-polyacrylamide gel. The electrophoresed proteins were transferred onto nitrocellulose membranes, and the membranes were subsequently incubated with the appropriate primary antibodies specific for each protein. Following reaction with the primary antibodies, the membranes were further incubated with secondary anti-immunoglobulin-G-horseradish peroxidase conjugates. Protein bands were visualized on X-ray film using an enhanced chemiluminescence system (Kodak). The β-actin expression was used as the reference band. The protein expression rate was quantified by Quantity One software.

### Statistical Analysis

All measurements were performed in triplicate, and all values are expressed as the mean ± standard error of the mean. The results were subjected to an analysis of variance and Tukey’s *post hoc* test to analyze differences between means. In each case, a *p*-value < 0.05 was considered statistically significant.

## Results

### Protective Effect of Cyanidin-3-*O*-Glucoside against UVB-Induced Cell Activity Decrease in HaCaT Cells

**FIGURE 1:**
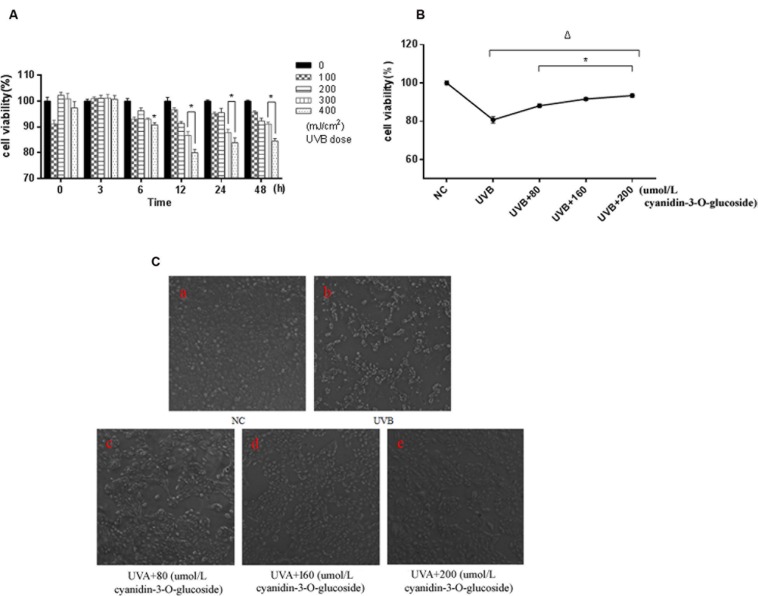
**Protective effect of cyanidin-3-*O*-glucoside against UVB-induced cell activity decrease in HaCaT cells. (A)** The effect of UVB on the activity of HaCaT cells. Results are presented as the mean ± standard error of the mean (%), *n* = 5; ^∗^*p* < 0.01. **(B)** Effect of cyanidin-3-*O*-glucosideon the activity of HaCaT cells after UVB irradiation. Results are presented as the mean ± standard error of the mean (%), *n* = 5; ^Δ^*p* < 0.01 vs NC, ^Δ^*p* < 0.01 vs UVB. **(C)** Effect of cyanidin-3-*O*-glucoside on the morphology of HaCaT cells after UVB irradiation (200×).

Ultraviolet B exposure inhibited the survival of HaCaT cells in a dose- and time-dependent manner, and the survival rate of the cells was significantly decreased for treatment with 300 mJ/cm^2^ UVB for 12 h (*p* < 0.01) (**Figure 1A**). However, the survival rate of the cells was elevated when cyanidin-3-*O*-glucoside was added after 1 h. Compared with the UVB-irradiated group, the three groups with cyanidin-3-*O*-glucoside showed significantly increased cell survival rates (*p* < 0.01) (**Figure 1B**). Cell morphology was observed using an inverted microscope (**Figure 1C**). Compared to the control group, 300 mJ/cm^2^ of UVB irradiation significantly reduced the number of cells, showed noticeable cell shrinkage, and decreased cell confluence, indicators of cellular stress. After the addition of different concentrations of cyanidin-3-*O*-glucoside, the cell morphology was restored, the number of cells increased, and cells showed greater confluence, but pre-irradiation characteristics were not restored.

### Scavenging Activity of Cyanidin-3-*O*-Glucoside against UVB-Generated Intracellular ROS in HaCaT Cells

**FIGURE 2:**
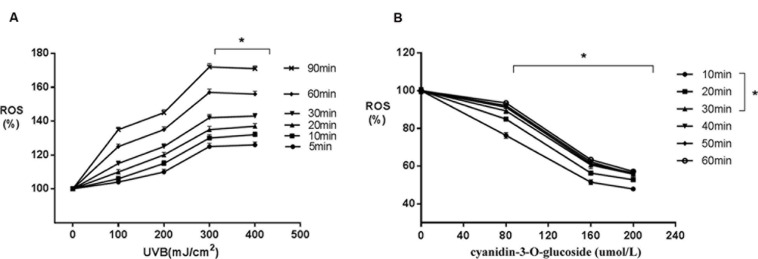
**Scavenging activity of cyanidin-3-*O*-glucoside against UVB-generated intracellular ROS in HaCaT cells. (A)** UVB induces intracellular ROS. Results are presented as the mean ± standard error of the mean (%), *n* = 4; ^∗^*p* < 0.01. **(B)** Cyanidin-3-*O*-glucoside scavenges intracellular ROS. Results are presented as the mean ± standard error of the mean (%), *n* = 4; ^∗^*p* < 0.01.

Increased UVB radiation doses induced gradual increases in ROS generation (**Figure 2A**). However, treatment with increasing concentrations of cyanidin-3-*O*-glucoside increased intracellular ROS scavenging in HaCaT keratinocytes (**Figure 2B**).

### Cytoprotective Effect of Cyanidin-3-*O*-Glucoside against UVB-Induced Apoptosis

**FIGURE 3:**
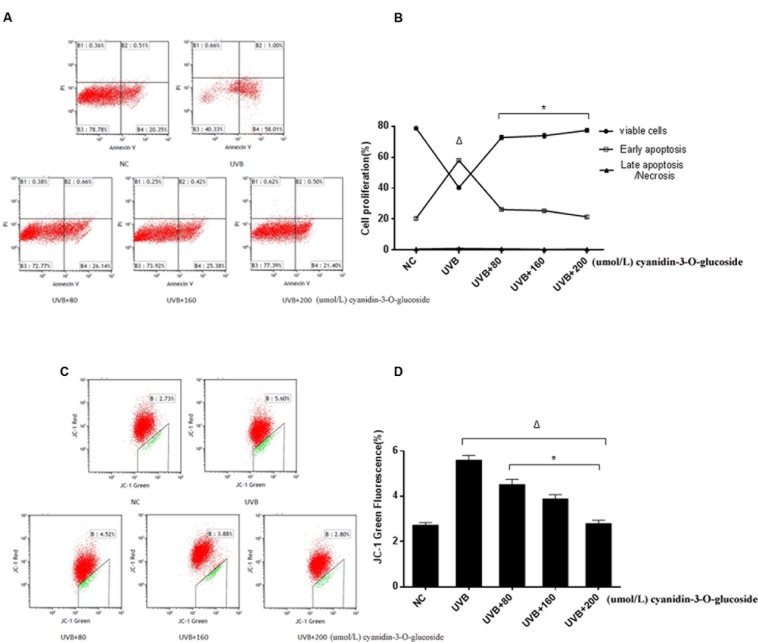
**Cytoprotective effect of cyanidin-3-*O*-glucoside against UVB-induced apoptosis. (A)** Flow cytometry results of apoptosis. **(B)** Apoptotic rate of HaCaT cells. Results are presented as the mean ± standard error of the mean (%), *n* = 3; ^Δ^*p* < 0.01, ^∗^*p* < 0.01 vs UVB. **(C)** Flow cytometry results of mitochondrial membrane potential (Δψm). **(D)** Δψm damage rate of HaCaT cells. Results are presented as the mean ± standard error of the mean (%), *n* = 3; ^Δ^*p* < 0.01 vs NC, ^∗^*p* < 0.01 vs UVB.

The effects to apoptosis were detected by flow cytometry and shown in **Figures 3A,B**. 80, 160, and 200 μmol/L cyanidin-3-*O*-glucoside reduced UVB-induced apoptosis from 58.01 to 25.38%, 26.14 and 21.40%, respectively, whereas the low level of necrosis showed no obvious change (*p* > 0.05) (**Figures 3A,B**). We next evaluated the mitochondrial membrane potential (Δψm) by staining with the membrane-permeable dye JC-1. The depolarized membrane regions of UVB-exposed cells were clearly observed as green fluorescence of JC-1 monomers, but the fluorescence intensity decreased after cyanidin-3-*O*-glucoside treatment (**Figures 3C,D**).

### Effects of Cyanidin-3-*O*-Glucoside on Apoptosis-Related Protein Expression in HaCaT Cells

**FIGURE 4:**
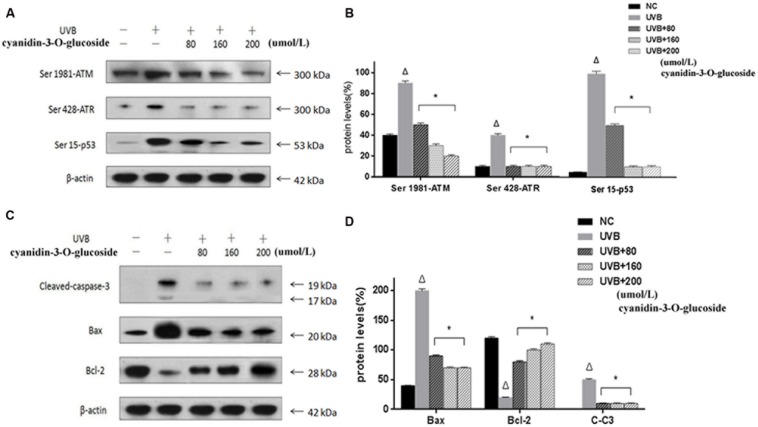
**Effects of cyanidin-3-*O*-glucosideon expression of apoptosis-related proteins in HaCaT cells. (A)** Western blot results for phospho-ATM/ATR and p53. **(B)** Phospho-ATM/ATR and p53 expression levels in HaCaT cells. **(C)** Western blot results for Bcl-2, Bax, and cleaved caspase-3. **(D)** Bcl-2, Bax, and cleaved caspase-3 expression levels in HaCaT cells. Results are presented as the mean ± standard error of the mean (%), *n* = 3; ^Δ^*p* < 0.01 vs NC, ^∗^*p* < 0.01 vs UVB.

ATM and ATR, the major kinases of the core molecular sensor, are recruited in response to DNA damage (Sancar et al., 2004), which is accompanied by activation of down-stream signaling molecules, finally resulting in cell cycle arrest or apoptosis. Therefore, ATM and ATR are commonly used to assess DNA damage in the cell. As expected, treatment of cells with UVB causes obvious DNA damage as indicated by up-regulation of the phosphorylation levels of ATM (Ser 1981), ATR (Ser 428), and p53 (Ser 15). In contrast, cyanidin-3-*O*-glucoside treatment strongly decreased the phosphorylation levels of ATM, ATR, and p53 (**Figures 4A,B**). We further investigated the expression levels of the anti-apoptotic protein B-cell lymphoma 2 (Bcl-2) and the pro-apoptotic proteins Bcl-2-associated X protein (Bax) and cleaved caspase-3. The expression of Bcl-2 was attenuated in UVB-exposed cells but restored in UVB/cyanidin-3-*O*-glucoside-treated cells. The expression of Bax was increased in UVB-irradiated cells and decreased in UVB/cyanidin-3-*O*-glucoside-treated cells. Moreover, the expression of cleaved caspase-3, a key executer of apoptosis, was also decreased by cyanidin-3-*O*-glucoside treatment of UVB-exposed cells (**Figures 4C,D**).

## Discussion

Ultraviolet light commonly consists of radiation of three wavelengths: UVA radiation, UVB radiation, and UVC radiation. Herein, we focused on the effects of UVB radiation, which is absorbed in greater amounts by human keratinocytes than UVA or UVC radiation (Liebel et al., 2012). The electron transport chain in the inner mitochondrial membrane, which is required for the major functions of these organelles, is also affected by UVB. Inhibition of electron transport not only leads to mitochondrial membrane depolarization, a decrease in mitochondrial oxygen uptake, and reduced phosphorylation of ADP to generate ATP, but also leads to an increase in ROS production following the incomplete reduction of molecular oxygen (O_2_) (Paz et al., 2008). Outer membrane-localized JC-1 monomers emitted strong fluorescence in UVB-treated keratinocytes, indicative of mitochondrial injury and oxidative stress (**Figure 3C**).

In the present study, we found that UVB can lead to changes in the morphology of human keratinocytes, and higher levels of ROS and higher apoptosis rates were induced by increasing UVB doses in a certain range (**Figures 1**–**3**), which was consistent with precious studies (Paz et al., 2008; Verschooten et al., 2010). UVB-induced DNA damage is particularly notorious for its association with apoptotic cell death because cells with lethal or irreparable damage to nuclear DNA are removed by apoptosis to limit the incidence and propagation of defective cells (Vostalova et al., 2010). Phospho-ATM/ATR and phospho-p53 are usually labeled as DNA damage response proteins in the cell. Herein, the expressions of these proteins were considerably augmented by UVB radiation in human HaCaT keratinocytes (**Figure 4**), confirming that UVB alters expression of DNA damage-associated proteins.

Recently, many research groups have searched for herbal compounds and extracts with a protective effect against UVB-induced oxidative stress. One of these compounds, cyanidin-3-*O*-glucoside, is an anthocyanin, phenolic pigments belonging to the flavonoid family that are known for their antioxidative, anticancer, and anti-inflammatory properties (Esposito et al., 2015). Anthocyanin-rich extracts can be obtained from purple corn, black soybean, blueberry, chokeberry, purple sweet potato, mulberry, cherry, grape, or black currant (Prior et al., 2008; Hogan et al., 2010). Due to the unique molecular structure and the large number of phenol hydroxyl groups (-OH) on cyanidin-3-*O*-glucoside, it has very strong antioxidant and free radical scavenging activities (Bailey et al., 2000). Here, we have showed that cyanidin-3-*O*-glucoside pretreatment decreased JC-1 fluorescence in irradiated cells and decreased the levels of intracellular ROS generated by UVB treatment (**Figures 2** and **3**). Western blotting analysis further showed that cyanidin-3-*O*-glucoside increased expression of anti-apoptotic factors, such as Bcl-2, in UVB-stressed cells, while simultaneously decreased the expression of pro-apoptotic factors, such as Bax and cleaved caspase-3 (**Figure 4**).

Murapa et al. (2012) previously showed that anthocyanin-rich fractions of blackberry extracts reduce UV-induced free radicals and oxidative damage in keratinocytes. However, although up-regulation of the expressions of catalase, MnSOD, Gpx1/2, and Gsta1 antioxidant enzymes were observed, the authors did not report the specific mechanism of apoptosis prevention and the protective effect. In our study, we found that cyanidin-3-*O*-glucoside inhibits ROS production to reduce UVB-evoked DNA damage and p53 levels, decrease the loss of mitochondrial membrane potential, and prevent apoptosis in keratinocytes. However, more detailed mechanisms of the intervention pathway of cyanidin-3-*O*-glucoside still need to be further studied.

## Conclusion

Cyanidin-3-*O*-glucoside has the capacity to protect human HaCaT keratinocytes from excessive ROS generated by UVB exposure and a protective effect against UVB-provoked DNA damage and apoptosis. Thus, cyanidin-3-*O*-glucoside may be useful as a therapeutic agent to mitigate the effects of excessive sun exposure and the ensuing oxidative stress.

## Author Contributions

The authors of YH, YM, and SW are responsible for the design of experiment, the experiment operation and writing the paper. The authors of TC, RJ, YH, and XJ are responsible for assist the experimental design, and a modification of the paper. The authors of JS and YH are responsible for extraction and separation of the cyanidin-3-*O*-glucoside. The authors of LD and WB guide the design of experiment and writing the paper.

## Conflict of Interest Statement

The authors declare that the research was conducted in the absence of any commercial or financial relationships that could be construed as a potential conflict of interest.
